# Effect of Polymer Addition on Performance of Portland Cement Mortar Exposed to Sulphate Attack

**DOI:** 10.3390/ma13010071

**Published:** 2019-12-22

**Authors:** Paweł Łukowski, Dominika Dębska

**Affiliations:** 1 Department of Building Materials Engineering, Faculty of Civil Engineering, Warsaw University of Technology, 00-637 Warsaw, Poland; 2 Chair of Building Materials Engineering, Faculty of Civil Engineering, Cracow University of Technology, 31-155 Cracow, Poland; ddebska@pk.edu.pl

**Keywords:** durability, polymer-cement composite, sulphate aggression

## Abstract

Resistance to degradation contributes greatly to the durability of materials. The chemical resistance of polymer-cement composites is not yet fully recognized. The goal of the research presented in this paper was to assess the performance of polymer-cement mortars under sulphate aggression, as compared to unmodified cement mortar. Mortars with polymer-to-cement ratios from 0 to 0.20 were stored in either a 5% MgSO_4_ solution or distilled water for 42 months. During this time, changes in elongation, mass, and compressive strength were determined. The results of these investigations, together with the visual and microscopic observations, allowed us to conclude that polymer–cement composites demonstrated better resistance to the attack of sulphate ions than unmodified cement mortar, even when using Portland cement with enhanced sulphate resistance.

## 1. Introduction

Cement composites, such as mortar and concrete, are relatively cheap but not very durable materials. The repair or replacement of these composites during exploitation of the building structure is technically difficult and usually expensive [1]. The durability of the building objects and materials, defined as the ability of the product to maintain its required performance over time [2], is an important element of sustainable development in construction [1].

One of the main reasons for the insufficient durability of concrete or mortar is degradation caused by chemical, physical, or biological factors. A durability assessment is possible using tests and observations of the material being exposed to the corrosive environment for a long time. Due to the lack of such data for the relatively recently introduced materials, various procedures are employed to accelerate ageing, compensating for the shorter time of investigation by the intensity of the corrosive factors.

Sulphate ions SO_4_^2–^ can cause substantial damages in the concrete. Sulphate corrosion is a complex process. First, a gypsum dihydrate is formed:

(1) $${\mathrm{Ca}(\mathrm{OH})}_{2}{\;+\;\mathrm{SO}}_{4}^{2-}\;\to {\;\mathrm{CaSO}}_{4}{\;+\;2\mathrm{OH}}^{-}$$

(2) $${\mathrm{CaSO}}_{4}{\;+\;2H}_{2}O\;\to {\;\mathrm{CaSO}}_{4}\cdot {2H}_{2}O$$

Then trisulphate (AFt, ettringite) is created from unreacted tricalcium aluminate:

(3) $$3\mathrm{CaO}\cdot {\mathrm{Al}}_{2}O_{3\;}+\;3\left({\mathrm{CaSO}}_{4}\cdot {2H}_{2}O\right){\;+\;26H}_{2}O\;\to \;6\mathrm{CaO}\cdot {\mathrm{Al}}_{2}O_{3}\cdot {3\mathrm{SO}}_{3}\cdot {32H}_{2}O$$

or from monosulphate (AFm):

(4) $$4\mathrm{CaO}\cdot {\mathrm{Al}}_{2}O_{3}\cdot {\mathrm{SO}}_{3}\cdot {12H}_{2}O\;+\;3\left({\mathrm{CaSO}}_{4}\cdot {2H}_{2}O\right){\;+\;14H}_{2}O\;\to \;6\mathrm{CaO}\cdot {\mathrm{Al}}_{2}O_{3}\cdot {3\mathrm{SO}}_{3}\cdot {32H}_{2}O.$$

These reactions are accompanied by significant stresses, leading to the weakening and damage to the structure of the material [3]. Sulphate corrosion of cement concrete has been a subject of many studies. According to Heinz and Ludwig [4], the main reason for the excessive internal stresses is the creation of the shells made from large, densely packed ettringite crystals around the aggregate grains. According to Brown and Taylor [5], the source of these stresses is the formation of nanometric ettringite crystals during the C-S-H phase [5]. Yu et al. [6] reported that in some cases gypsum is formed in the later stages of deterioration and thus its presence does not necessarily mean the beginning of the concrete degradation. The pressure caused by the growth of the ettringite crystals is commonly accepted as the most probable mechanism of concrete microstructure deterioration [7,8].

However, not all of the questions have already been answered. For instance, the effects of the simultaneous attack of sulphate ions and other corrosive factors (e.g., freezing and thawing cycles [9] or mechanical loading [10]) need to be considered. A model of the concrete deterioration under sulphate attack has been developed [11], as has a model of sulphate ion transport in concrete [12]. Another field of research in this area is the impact of various modifiers on the concrete’s resistance to sulphate aggression. In this context, the results of nanomodification [13] and the effects of industrial wastes in the concrete are studied [14].

Polymers are one widely used modifiers of contemporary concrete. By adding polymers to the cement concrete or mortar mix, polymer-cement composites (PCCs) are created. PCC materials belong to the group of building polymer composites, together with resin concretes and mortars and polymer-impregnated concretes [15,16]. The polymer in such a composite is a co-binder [17], creating an interpenetrating network with Portland cement. Polymer-cement concretes and mortars are successfully used in construction; one example of their application is the repair and protection of concrete structures, making pavements (including industrial floors), and the production of pre-cast elements [18]. Polymer-cement composites demonstrate substantial advantages over ordinary concrete and mortar regarding properties such as tensile and flexural strength, adhesion to various substrates, and tightness [19,20,21]. They also have a significant potential for efficient utilization of burdensome industrial wastes since it has been demonstrated that such materials as various types of fly ashes and waste perlite powder can be successfully used as components of polymer-cement composites [22]. However, the question of PCC durability has not yet been fully resolved. The majority of published papers show a rather optimistic view of this problem, based on the published test results [23,24,25]. However, there are other issues that need to be considered. One of these is the resistance of polymer–cement composites to chemical attacks.

This paper presents our investigation of the impact of polymer addition on PCC resistance to sulphate attack. The experiment compared the behaviour of polymer-cement and ordinary cement mortars exposed to sulphate aggression. Polyacrylic ester (PAE) in the form of water dispersion (solid content 54% ± 1%) was used as a polymer co-binder.

The improvement of Portland cement composite performance by polymers is a result of two basic mechanisms [15]. The first is the strengthening of the aggregate-paste interfacial transition zone, which is the most porous and weakest place in ordinary concrete or mortar. In PCCs, however, the voids are filled with polymer particles that also increase the adhesion between the aggregate grains and the cement matrix. The second mechanism is the crack-bridging ability of the polymer. Generally, since polymers are commonly used as modifiers for the cement composites (i.e., PAEs, styrene-acrylic copolymers, styrene-butadiene rubber, ethylene-vinyl acetate copolymer, polyvinyl alcohol, and epoxy resins), these mechanisms do not depend on the chemical nature of the polymer [21]. Therefore, the choice of the polymer modifier is based mainly on the technological ease of its application, availability, and economic rationality. The PAEs are disperse well in water and have a favourable low minimum film-forming temperature of about 0 °C [18]. For this reason, they are one of the most often used polymer modifiers and, simultaneously, their effect on the performance of cement composites is representative of the wide group of polymer modifiers.

## 2. Materials and Methods

The following materials were used to prepare the tested mortars:

- Portland cement CEM I 42.5 N according to European Standard EN 197-1 [26], with enhanced resistance to sulphate corrosion (medium sulphate resistance, MSR). The chemical composition of the cement is presented in Table 1.
- CEN standard sand 0-2 mm according to European Standard EN 196-1 [27].
- Tap water, conforming to European Standard EN 1008:2004 [28].
- A commercially available polymer modifier: a PAE in the form of water dispersion (solid content 54%) (MC-Bauchemie, Sroda Wlkp., Poland). The basic properties of the polymer are presented in Table 2.

The use of sulphate-resistant cement was chosen based on the main aim of the research, which was the verification of the hypothesis that the polymer addition can further improve the sulphate resistance of the composite, even if it already contains sulphate-resistant cement.

The reference material was not modified (polymer-to-cement ratio, p/c = 0): standard Portland cement mortar according to European Standard EN 196-1 [27] (the cement-to-sand ratio was 1:3 and water-to-cement ratio was 1:2). The polymer-cement ratio in polymer-cement mortars was 1:20, 1:10, and 1:5 (by mass). The mix compositions are presented in Table 3.

The beam specimens with dimensions 2 cm × 2 cm × 16 cm were cast for all proposed mixes, according to the European Prestandard ENV 196-X [29]. All specimens were cured for 28 days. The cement mortar specimens were stored in water, while polymer-cement mortar specimens were stored for 7 days in water and later kept in standard laboratory conditions (temperature 20 ± 2 °C and relative humidity 60% ± 10%, according to the Warsaw University of Technology procedure for PCC curing [30]). Half of the specimens were placed in a 5% MgSO_4_ solution (the solution was replaced every 28 days to keep the concentration constant), and the second half were stored in distilled water. The temperature of both media was 20 ± 2 °C. The tested properties included elongation and mass changes as well as compressive strength. The measurements were performed after 28 days of curing (i.e., immediately before placing the specimens in the sulphate solution or distilled water) and then after 6, 12, 18, 30, and 42 months of storing the specimens in the sulphate solution or distilled water. Specimens of each mortar type, stored in both media, were measured with the accuracy of 0.01 mm (with a Graf-Kaufman apparatus, EMEL, Warsaw, Poland, according to the prENV 196-X [29]) and weighed (after 24 h of drying in standard laboratory conditions) with the accuracy of 0.01 g. The specimens were cut in half and used for the compressive strength determination (according to EN 197-1 [26], with only deviation referring to the size of specimens). The length and mass of the specimens were controlled during each replacement of the sulphate solution. Some visual and microscopic observations were also conducted.

Magnesium sulphate(VI) solution was selected as the corrosive medium because MgSO_4_ is harmful to all cement minerals, including the C-S-H phase, while sodium sulphate, according to some studies [31], attacks mainly portlandite and tricalcium aluminate. Thus, strength reduction can be determined earlier when magnesium sulphate(VI) is used as the aggressive factor.

## 3. Results and Discussion

This investigation covered the determination of elongation and mass changes, as well as the compressive strength of the specimens made of Portland cement mortars which contained different amounts of a polymer modifier, PAE (polymer-to-cement ratio 0–0.20 by mass) after various times of exposure to either a 5% MgSO_4_ solution or distilled water (0–42 months).

### 3.1. Elongation

**Figure 1:**
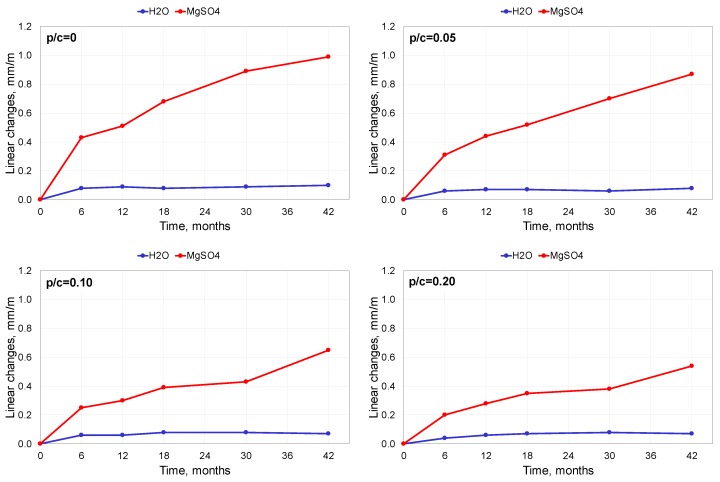
Elongation of mortar specimens exposed to 5% MgSO_4_ solution and distilled water.

The elongation of the specimens is presented in Figure 1.

The changes in length of the specimens stored in water were almost negligible, independent of the level of polymer addition. However, when exposed to the 5% MgSO_4_ solution, the mortar specimens showed elongation that increased with the time of exposure. The changes were significant in spite of using Portland cement with enhanced sulphate resistance (MSR). The polymer addition improved mortar performance in such circumstances. The analysis of the results showed that the elongation diminished with increasing polymer content.

**Figure 2:**
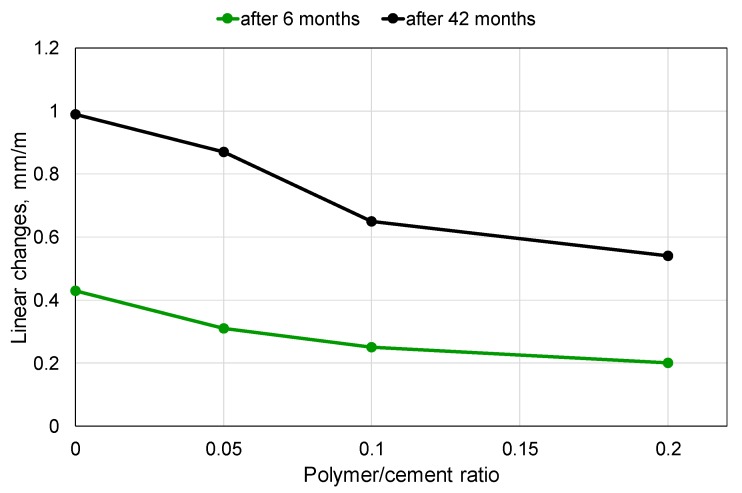
Elongation of mortar specimens with various content of polymer modifier after 6 and 42 months of exposure to the 5% MgSO_4_ solution.

The relative differences in elongation between the mortar with the polymer-cement ratio of 0.20 and that containing no polymer were similar for various times of exposure, and were roughly close to 50%. For instance, after 6 months of exposure, the absolute difference was equal to 0.23 mm/m, which meant a decrease by 53%, while after 42 months, the absolute difference was equal to 0.45 mm/m, which meant a decrease by 45.5% (Figure 2).

### 3.2. Mass Changes

**Figure 3:**
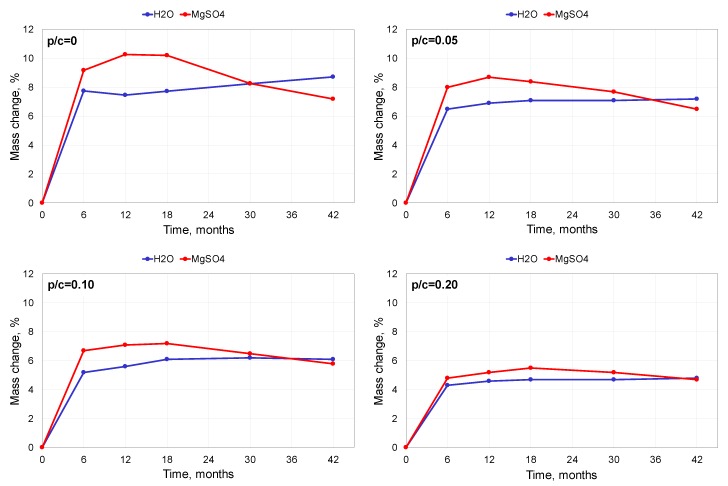
Mass changes of mortar specimens exposed to the 5% MgSO_4_ solution and distilled water.

**Figure 4:**
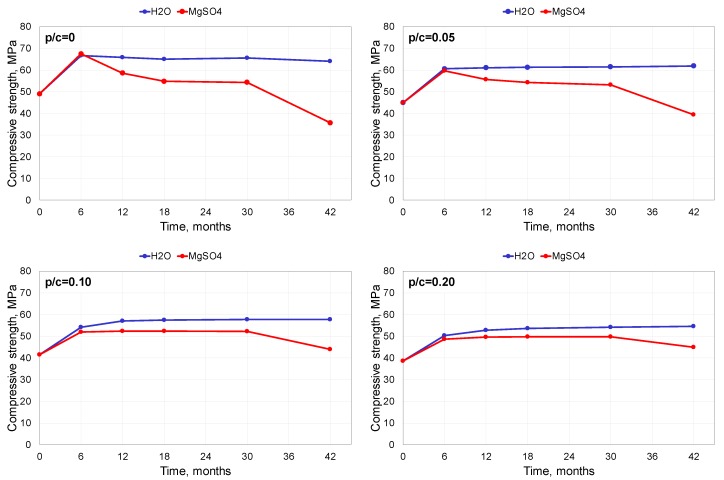
Compressive strength of mortars exposed to 5% MgSO_4_ solution and distilled water.

The results of the mass change determination are presented in Figure 3. The mass changes of the specimens stored in water were caused by water absorbance. After a significant increase within the first 6 months, it stabilized to some extent. The addition of polymer diminished the water absorbability of the mortars to a greater degree as the polymer content increased. This was due to the changes in the porosity structure: at a similar level of the total pores volume, the presence of polymer led to the domination of finer and closed pores. Additionally, the formed polymer film is an obstacle for the moisture transport inside the polymer-cement composite. The behaviour of the specimens exposed to the 5% MgSO_4_ solution was different. The initial increase of the mass was higher than that in water as it was caused not only by water absorbance but also by forming additional substances inside the specimens—gypsum and ettringite, the products of sulphate corrosion of the cement composites. However, after a longer exposure time, the mass of the specimens began to decrease. This was a result of damage to the specimens caused by the deterioration of the internal structure of the mortar by the corrosion products. The damage could be observed mainly in the form of corners and edges spalling (see the observations presented in Section 3.4.). This negative effect was mitigated by the polymer and the degree of this improvement increased with the polymer content. This phenomenon can be explained by the fact that the water is necessary for the chemical reactions causing deterioration of the composite, and because the presence of polymer reduces the water absorbability, as mentioned above. In a few cases, the values of mass changes presented in Figure 4 for the specimens stored in water and in the sulphate solution seem to be close, but the standard deviations of the average values are low enough (below 1% in either case) to confirm the reliability of the observed tendencies.

### 3.3. Compressive Strength

The results of the compressive strength investigation are presented in Figure 4.

The development of the strength of the mortars stored in water was typical for cement composites. The compressive strength increased for the first 6 months of storage, then stabilized at a relatively high level. The compressive strength of polymer-cement mortars increased for a longer period of time but remained lower in value than that of the unmodified composite. This is a well-known phenomenon, caused by disruption of the cement hydration by an otherwise advantageous polymer film [15]. The continuous polymer film hinders the moisture transport inside the composite. This leads to a deficiency of the water necessary for the cement hydration process and thus reduces the rate of hydration. For this reason, the strength development in PCCs is usually delayed as compared to unmodified composites [17,21].

Corrosion processes in the mortar attacked by sulphate ions led to a deterioration of the strength. The compressive strength of the unmodified mortar exposed to the 5% MgSO_4_ solution developed until the sixth month of storage, then began to decrease; the decrease was particularly noticeable after 30 months of exposure. The compressive strength decreased from almost 70 MPa after 6 months to less than 40 MPa after 42 months of storage.

The polymer addition improved the performance of the cement mortar exposed to the sulphate solution. At the polymer-cement ratio of 0.05, the decrease of the strength was much milder than in the case of the unmodified mortar. At higher contents of the polymer modifier, the compressive strength did not decrease after 6 months of exposure, and only after the 30th month could some diminishment of the strength be observed.

**Figure 5:**
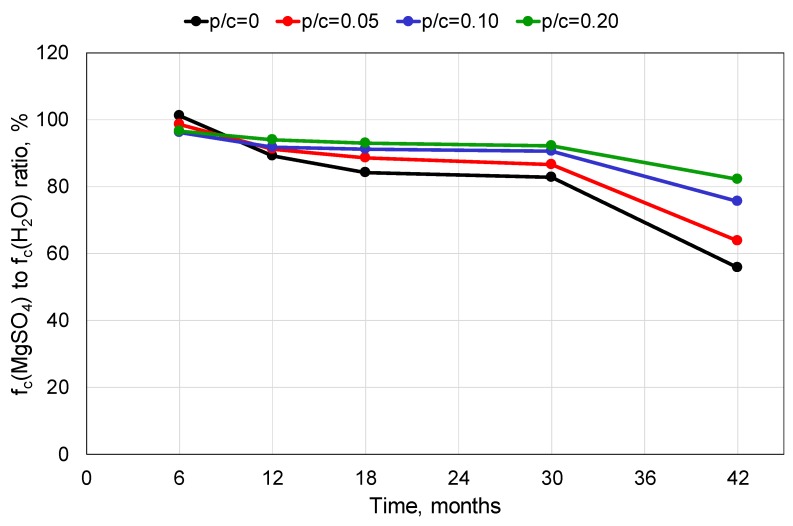
The ratio of compressive strength of mortars stored in 5% MgSO_4_ solution to those stored in distilled water after different exposure times, for various polymer contents.

Figure 5 presents the ratio of the compressive strength of the mortars exposed to the 5% MgSO_4_ solution to that of the mortars stored in pure water, for the range of exposure times. It clearly demonstrates that an increase in the polymer content led to a better performance of the composite. For the unmodified mortar, the loss of the compressive strength after 42 months of exposure to the sulphate solution was 44% compared to that determined for the mortar stored in water. For the mortar with the polymer-cement ratio of 0.20 it was only 18%.

### 3.4. Visual and Microscopic Observations

The visual observations generally corresponded with the results of the technical properties investigations.

**Figure 6:**
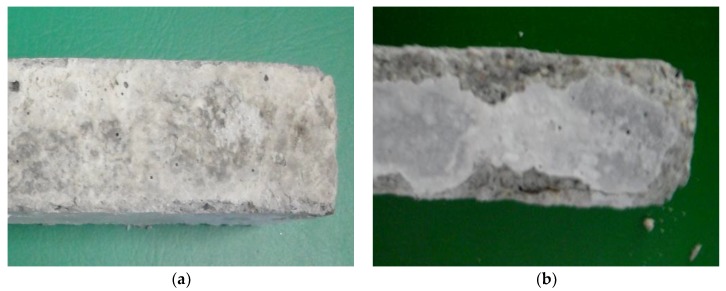
Cement mortar without polymer stored in 5% MgSO_4_ solution: (**a**) after 12 months; (**b**) after 42 months.

**Figure 7:**
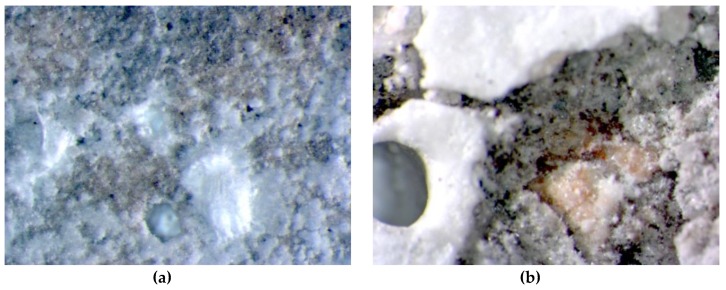
Microscopic images of cement mortar without polymer stored in 5% MgSO_4_ solution: (**a**) after 12 months; (**b**) after 42 months; magn. 50×.

The unmodified Portland cement mortar began to demonstrate clear signs of corrosion after 12 months of exposure to the 5% MgSO_4_ solution (Figure 6a). After 42 months of exposure, the specimens were seriously damaged, which is in good correlation with the results of previously reported test results (Figure 6b). In addition, the structure of the mortar was damaged in a manner corresponding to the above observations. Needle-like ettringite crystals were present after 12 months (Figure 7a), while—as described in the Introduction—massive ettringite forming the shells around the aggregate grains, dominated after 42 months of exposure (Figure 7b).

**Figure 8:**
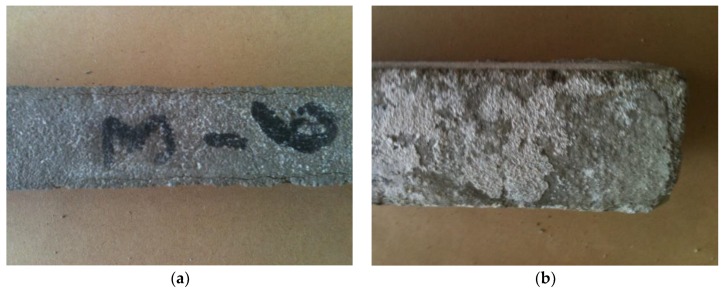
Polymer-cement mortar, p/c = 0.05, stored in 5% MgSO_4_ solution: (**a**) after 12 months; (**b**) after 42 months.

**Figure 9:**
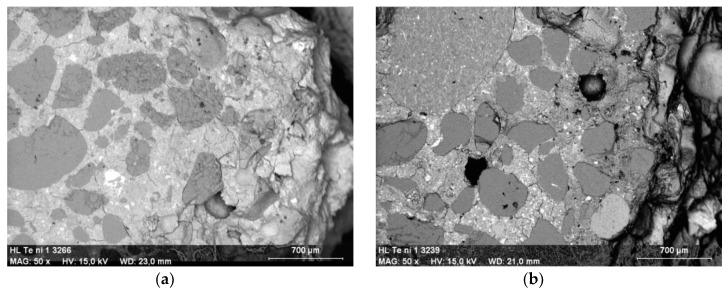
Microscopic images of polymer-cement mortar, p/c = 0.05, stored in 5% MgSO_4_ solution: (**a**) after 12 months; (**b**) after 42 months.

Adding polymer to the Portland cement mortar led to an improvement in the composite performance subjected to the sulphate aggression. Even a small content of the polymer modifier (polymer-cement ratio 0.05) delayed the beginning of corrosion (Figure 8a). However, after a longer exposure, this composite showed a similar degradation as that without modification (Figure 8b). Due to the action of the polymer, the structure of the composite was more homogeneous and compacted, thus less water-permeable (Figure 9a). However, after 42 months, clear signs of weakening of the structure could be seen, namely microcracks and delaminations in the aggregate-paste contact zone (Figure 9b).

**Figure 10:**
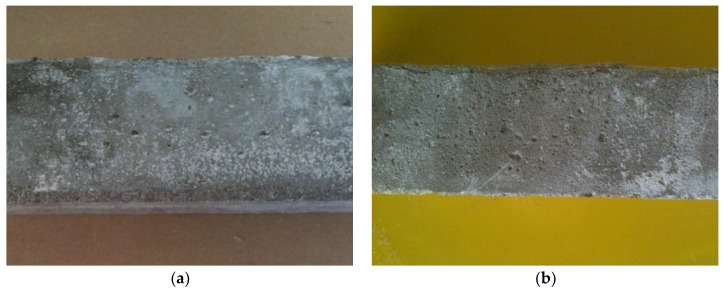
Polymer-cement mortar, p/c = 0.20, stored in 5% MgSO_4_ solution: (**a**) after 12 months; (**b**) after 42 months.

**Figure 11:**
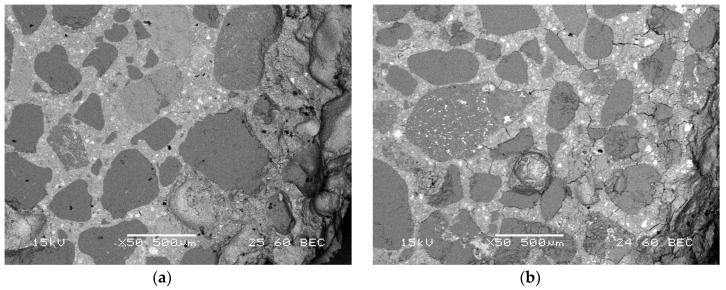
Microscopic images of polymer-cement mortar, p/c = 0.20, stored in 5% MgSO_4_ solution: (**a**) after 12 months; (**b**) after 42 months.

Higher polymer content brought more substantial enhancement in the mortars’ behaviour. After 12 months of exposure to the 5% MgSO_4_ solution, only efflorescence without advanced corrosion symptoms could be found on the specimens’ surfaces (Figure 10a), while after 42 months of exposure, the specimens were still in satisfactory shape, with only moderate visible signs of corrosion (Figure 10b). The microscopic observations of the structure of the polymer-cement mortars were in good correlation to these findings. The structure of the composite with a polymer-cement ratio of 0.20 looked sound after 12 months of exposure to the sulphate solution (Figure 11a), while after 42 months of exposure, it showed only slight microcracks and deformations (Figure 11b). The action of the polymer content was sufficient to ensure not only a tighter structure but also better adhesion between aggregate grains and hardened cement paste.

## 4. Conclusions

The results of investigations presented in this paper allow us to conclude that the addition of the polymer modifier to the Portland cement mortar could greatly improve its performance when exposed to sulphate aggression. The measurements of elongation and mass changes of the specimens, as well as the determination of the compressive strength, showed a significant advantage of the polymer-cement composite over the unmodified one regarding their behaviour during long-term (up to 42 months) storage in a 5% magnesium sulphate(VI) solution. In these conditions, the elongation of PCCs with a polymer-cement ratio of 0.20 was about 50% of that measured for the mortar without polymer. The mass of the tested mortar specimens stored in water stabilized when they achieved saturation, however, the mass of the specimens stored in the sulphate solution started to decrease after 12 to 18 months due to damage to the specimens. The polymer mitigated this effect, reducing the absorbability of the water necessary for the reactions that lead to damage. For PCCs with a polymer-cement ratio of 0.20, the mass increase after 42 months of storage in the sulphate solution was very close to that in water and did not exceed 5%. Determination of the compressive strength also showed a significant advantage of the polymer-cement composite over the unmodified one. The compressive strength of the unmodified mortar exposed to the 5% MgSO_4_ solution decreased from almost 70 MPa after 6 months to less than 40 MPa after 42 months of storage. The compressive strength of PCCs with a polymer-cement ratio of 0.20 after 6 months of storage was about 50 MPa, but after 42 months it still exceeded 45 MPa. For the unmodified mortar, the loss of the compressive strength after 42 months of exposure to sulphate solution was 44% compared to that determined for the mortar stored in water, while for the mortar with the polymer-cement ratio of 0.20 it was only 18%. Within the polymer-cement ratio range from 0 to 0.20, the resistance of the material to sulphate aggression improved as the polymer content increased. These findings are in good correlation with the results of the visual inspection of the tested specimens and microscopic observations.

## Figures and Tables

**Table 1:** Chemical composition of cement.

Component	Content, in mass %
CaO	64.89
SiO_2_	21.60
MgO	0.76
Al_2_O_3_	3.44
Fe_2_O_3_	3.50
SO_3_	2.71
Na_2_O_eq_	0.32
Cl	0.018
Insoluble part	0.38
Loss on ignition	1.43
C_3_A	3.20
C_3_S	59.9

**Table 2:** Basic properties of polyacrylic ester (PAE).

Property	Unit	Value
Form of delivery	-	Water dispersion
Solid content	%	54 ± 1
Density of dispersion	kg/m^3^	1270 ± 10
Minimum film-forming temperature	°C	~0

**Table 3:** Mixture compositions (in g).

Series	Cement	Water ^1^	Sand	Polymer
p/c = 0	450	225	1350	0
p/c = 0.05	450	225	1350	22.5
p/c = 0.10	450	225	1350	45.0
p/c = 0.20	450	225	1350	90.0

^1^ Including that contained in the polymer dispersion.
